# Mortality Statistics, Chicago

**Published:** 1875-08

**Authors:** 


***Chicago Mortality Report for June, 1875.*** Reported by Dr. BEN. C. MILLER, Sanitary Superintendent.

| | |
|---|---|
| Accident, crushed | 1 |
| " drowning | 8 |
| " explosion | 2 |
| " fall | 3 |
| " run over by wagon | 1 |
| " kicked by horse | 1 |
| " poison | 2 |
| " railroad | 1 |
| " street cars | 1 |
| " shooting | 1 |
| Abscess, lumbar | 1 |
| Anus, imperforate | 1 |
| Apoplexy | 11 |
| Atelectasis pulmonum | 1 |
| Bladder, inflammation of | 1 |
| Bowels, obstruction of | 1 |
| " hæmorrhage of | 1 |
| Brain, compression of | 1 |
| " congestion of | 8 |
| " disease of | 1 |
| " inflammation of | 10 |
| " softening of | 2 |
| Bronchitis | 5 |
| " capillary | 7 |
| Catarrh | 1 |
| Cancer | 1 |
| " of breast | 2 |
| " of stomach | 3 |
| " of uterus | 5 |
| Child birth | 2 |
| Cholera infantum | 33 |
| " morbus | 1 |
| Consumption | 48 |
| Convulsions | 62 |
| " puerperal | 2 |
| Croup | 6 |
| Cyanosis | 3 |
| Debility, general | 7 |
| Deficient development | 1 |
| Diarrhœa | 17 |
| " chronic | 1 |
| Diphtheria | 7 |
| Dropsy, general | 6 |
| Dysentery | 5 |
| Embolism | 1 |
| Endo carditis | 4 |
| Eczema | 1 |
| Enteritis | 9 |
| Entero colitis | 3 |
| Epilepsy | 1 |
| Erysipelas | 4 |
| Fever, congestive | 1 |
| " puerperal | 2 |
| " remittent | 1 |
| " scarlet | 15 |
| " typhoid | 4 |
| Gastritis | 2 |
| Hæmatemesis | 1 |
| Heart, disease of | 3 |
| " rheumatism of | 1 |
| " valvular disease of | 4 |
| Hepatitis | 1 |
| Hernia, incarcerated | 1 |
| Hydrocephalus | 12 |
| Inanition | 15 |
| Intemperance | 2 |
| Intussusception | 1 |
| Kidneys, Bright's disease of | 6 |
| " inflammation of | 2 |
| Liver, atrophy of | 1 |
| " disease of | 1 |
| " cirrhosis of | 1 |
| Lungs, congestion of | 2 |
| " emphysema of | 1 |
| " hæmorrhage of | 1 |
| " œdema of | 1 |
| " paralysis of | 1 |
| Manslaughter | 2 |
| Malformation | 1 |
| Metro peritonitis | 1 |
| Measles | 18 |
| Meningitis | 17 |
| " cerebro-spinal | 7 |
| " tubercular | 5 |
| Myelitis | 1 |
| Old age | 12 |
| Paralysis | 2 |
| Pericarditis | 1 |
| Peritonitis | 5 |
| " puerperal | 1 |
| Purpura | 1 |
| Pneumonia | 21 |
| " typhoid | 3 |
| Pleurisy | 1 |
| Pyæmia | 3 |
| Rheumatism | 1 |
| Scrofula | 3 |
| Scepticæmia | 1 |
| Small-Pox | 1 |
| Spine, disease of | 1 |
| Stone in bladder | 1 |
| Sun stroke | 2 |
| Suicide, by laudanum | 1 |
| " by drowning | 1 |
| " by hanging | 1 |
| Syphilis | 2 |
| Tabes mesenterica | 10 |
| Teething | 3 |
| Tetanus | 2 |
| Tedious birth | 1 |
| Trismus | 1 |
| Tumor, on neck | 1 |

| | | | |
|---|---|---|---|
| Tumor, shock from operation | I | Whooping cough | 4 |
| Vitality, deficient | I | | |
| | | Total | 533 |

Premature births, 9 ; Still births, 47. Total, 56.

COMPARISON.

Deaths in June, 1875, 533 ; in May, 1875, 603. Decrease, 70. Deaths in June, 1874, 542. Decrease, 9.

AGES.

| | | | |
|---|---|---|---|
| Under one year | 210 | Thirty years to forty | 48 |
| One year to two | 52 | Forty " " fifty | 41 |
| Two years to three | 21 | Fifty " " sixty | 23 |
| Three " " four | 8 | Sixty " " seventy | 18 |
| Four " " five | 7 | Seventy " " eighty | 14 |
| Five " " ten | 26 | Eighty " " ninety | II |
| Ten " " twenty | 23 | | |
| Twenty " " thirty | 34 | Total | 533 |

| | | | | | |
|---|---|---|---|---|---|
| White | 520 | Males | 288 | Married | 163 |
| Colored | 13 | Females | 245 | Single | 370 |
| Total | 533 | Total | 533 | Total | 533 |

NATIVITIES.

| | | | | | |
|---|---|---|---|---|---|
| Austria | 2 | Denmark | I | Norway | 6 |
| Belgium | I | England | 8 | Poland | 2 |
| Bohemia | 7 | France | I | Scotland | 3 |
| Canada | 5 | Germany | 61 | Sweden | 2 |
| Native—Chicago | 88 | Holland | 2 | Switzerland | 2 |
| Foreign, " | 204 | Ireland | 46 | Unknown | 3 |
| U. States, other parts | 87 | Italy | 2 | | |
| | | | | Total | 533 |

Deaths daily, 17¾. Mean thermometer, 63°. Rain fall, 5.17 inches.

MORTALITY BY WARDS.

| Wards. | No. Deaths. | Pop. in 1874. | Percentage. | Wards. | No. Deaths. | Pop. in 1874. | Percentage. |
|---|---|---|---|---|---|---|---|
| I | 2 | 5,725 | one death in 2,862 | II | 13 | 14,022 | one death in 1,078 |
| 2 | I | 4,830 | " " 4,830 | 12 | 15 | 16,792 | " " 1,119 |
| 3 | 18 | 14,861 | " " 826 | 13 | 13 | 17,892 | " " 1,376 |
| 4 | 9 | 15,361 | " " 1,707 | 14 | 17 | 16,720 | " " 983 |
| 5 | 22 | 20,078 | " " 913 | 15 | 103 | 45,545 | " " 442 |
| 6 | 42 | 35,916 | " " 855 | 16 | 24 | 21,922 | " " 913 |
| 7 | 33 | 31,722 | " " 961 | 17 | 29 | 20,777 | " " 717 |
| 8 | 36 | 29,143 | " " 809 | 18 | 27 | 21,392 | " " 792 |
| 9 | 42 | 31,654 | " " 754 | 19 | 7 | 4,677 | " " 668 |
| 10 | 15 | 17,385 | " " 1,126 | 20 | 10 | 8,995 | " " 899 |

Ratio of deaths to population in 1874, one death in 743⅞.

| | | | |
|---|---|---|---|
| No. deaths in Wards | 478 | Manslaughter | 2 |
| Accidents | 21 | Small-Pox Hospital | I |
| Bridewell | I | St. Joseph's Hospital | I |
| County Hospital | 6 | " " Orphan Asylum | I |
| Foundlings' Home | 16 | Suicides | 3 |
| Hospital Alexian Bros. | 2 | | |
| Mercy Hospital | I | Total | 533 |

